# The Exploration of the Urethra and Bladder

**Published:** 1900-03

**Authors:** 


**The Exploration of the Urethra and Bladder.** By M. Tuchmann, M.D. Pp. vi., 56. London: H. K. Lewis. 1899.

The author of this monograph has devoted many years of patient study and investigation to the subject of the anatomy of the human bladder, especially as affecting the use of catheters and sounds, and gives us the results of his observations in an interesting and practical manner. The principal object of his investigations seems to have been the perfecting of an instrument called "The Ureter Forceps," to be used for introduction into the bladder for the purpose of grasping and closing the orifice of one of the ureters so as to examine the urine coming from each kidney separately. Although the author tells us that he has used this instrument for the last twenty-five years, and has introduced it into the bladder of living subjects more than 1000 times, and has never had any bad results, yet we are not convinced of the harmlessness of the procedure. He recommends that the orifice of the ureteric valve should be grasped within the blades of the forceps and held closed for fifteen minutes, during which time the urine is allowed to flow from the other ureter only, so that it can be collected and examined. We fear that injury to the walls of the bladder must inevitably ensue in many cases as a result of this pinching up of the mucous coat, and we are of opinion that the use of the cystoscope is much more likely to be of service in this method of examining the urine of each kidney separately than the use of the ureter forceps, although, doubtless, the author by long experience and practice has rendered his employment of the instrument quite simple and safe.

The book is well worthy of perusal, as it is evidently the work of a lifetime of careful experimental research.

